# Efficient Separation and Enrichment of Rubidium in Salt Lake Brine Using High-Performance PAN-KCuFC-PEG Adsorption Composite

**DOI:** 10.3390/molecules30061273

**Published:** 2025-03-12

**Authors:** Linhong Wu, Kun Zhou, Yuchen Zheng, Ying Zeng, Guangyong Zeng, Ziyi Cheng, Yang Peng

**Affiliations:** School of Materials and Chemistry & Chemical Engineering, Chengdu University of Technology, Chengdu 610059, China; wulinhong@stu.cdut.edu.cn (L.W.); zhengyuchen28@stu.cdut.edu.cn (Y.Z.); zengy@cdut.edu.cn (Y.Z.); zengguangyong18@cdut.edu.cn (G.Z.); ziyicheng@stu.cdut.edu.cn (Z.C.); yangpeng2@stu.cdut.edu.cn (Y.P.)

**Keywords:** salt lake brine, rubidium, adsorption and purification, chromatography, polyethylene glycol

## Abstract

Salt lake brine contains abundant rubidium resources; however, the separation of rubidium from brine with a high K content remains a significant challenge in metallurgical processes and materials science. In this study, PAN-KCuFC-PEG particles were synthesized by phase transformation, using hydrophilic polyacrylonitrile (PAN) as the skeleton structure, potassium cupric ferricyanide (KCuFC) as the active component and water-soluble polymer polyethylene glycol (PEG) as the pore regulator. Characterization revealed that the addition of PEG increased the pore volume of PAN-KCuFC-PEG by 63% and the BET surface area by 172%. KCuFC powder was uniformly dispersed in PAN-KCuFC-PEG, and its crystal structure remained stable after loading. In static adsorption experiments, the maximum adsorption capacity of PAN-KCuFC-PEG for Rb^+^ reached 190 mg/g. The adsorption behavior followed a pseudo-second-order kinetic model, with the rate jointly controlled by external diffusion, intraparticle diffusion, and chemical reaction. In the column experiment, PAN-KCuFC-PEG was used to adsorb Qarhan Salt Lake brine (K: 26,000 mg/L, Rb: 65 mg/L). NH_4_Cl was employed for elution and desorption of PAN-KCuFC-PEG. During the adsorption–desorption process, the separation factor between Rb and K reached 160, the desorption rate reached 96.6%, and the overall yield was 68.3%. The enrichment and separation of Rb were successfully achieved.

## 1. Introduction

Rubidium, an industrial metal that is both scarce and costly, is of significant interest in a variety of fields [1]. Due to its distinctive physicochemical properties, rubidium finds wide application in electronic devices, medical treatment, atomic clocks, new energy sources, nuclear medicine, aerospace engineering, and numerous other fields [2,3,4,5]. The demand for rubidium has increased in tandem with the advancements in optoelectronic materials, atomic clocks, and laser technology, resulting in a steady rise in the price of rubidium salts annually. According to the United States Geological Survey (USGS), the price of 10 g of rubidium chloride reached USD 80.1 in 2023, marking a 20% increase compared to USD 67.10 in 2022.

The global supply of rubidium is substantial and geographically dispersed. However, the mineral is predominantly associated with lithium, beryllium, niobium, salt lake brine, and other rare metal minerals [6]. The paucity of ore resources, in conjunction with the complexity of extracting rubidium from ores, has precipitated the recovery of Rb^+^ from alternative water sources, including salt lakes, seawater, and their associated brines [6,7,8]. Typically, the rubidium content in brine is minimal, and there is a substantial presence of interfering ions, particularly the alkali metal ions Na^+^ and K^+^. These ions are analogous to rubidium in terms of their chemical properties, which complicate the separation and extraction processes [9].

A variety of chemical processes have been developed for this purpose, including precipitation [10], extraction [11], and ion exchange [12,13,14]. Ion exchange demonstrates its adaptability to brines featuring low rubidium concentrations while concurrently presenting a minimal environmental footprint, as detailed in reference [15]. The predominant ion exchangers for rubidium have been identified as oxides [16], heteropolymers [17], thiostannates [18] and aluminosilicates [19], as well as the metals ferrocyanide and ferricyanide [20]. Among the aforementioned compounds, potassium copper ferrocyanide (KCuFC) and its analogs have demonstrated remarkable capabilities in terms of Rb^+^ adsorption capacity and selectivity [21]. However, owing to the ultrafine particle size of KCuFC, it tends to aggregate readily. Additionally, it suffers from low mechanical strength and poses challenges in terms of separation from water. These characteristics collectively render it challenging to be directly implemented in actual production processes, as documented in reference [22]. The resolution of this issue entails the fabrication of composite adsorbents that are readily separable. This objective can be accomplished by loading KCuFC into the material using functionalized materials, such as minerals, silica, metal–organic skeletons, and resins, among others [23,24,25].

Polyacrylonitrile (PAN), a high-molecular-weight polymer known for its high mechanical strength and strong chemical stability, is widely used in the field of adsorption [26]. PAN-based ferrocyanide adsorbents have also been employed for rubidium enrichment in seawater [27]. Polyethylene glycol (PEG), utilized as a pore regulator, is extensively applied in membrane synthesis and adsorbent preparation to modulate pore structures and enhance adsorption performance. Studies indicate that KCuFC particle adsorbents supported by carboxymethyl cellulose (CMC) exhibit a cesium adsorption capacity of 60.8 mg/g [28]. Remarkably, the adsorption capacity of the CMC-KCuFC-PEG composite synthesized with PEG incorporation increases to 149.8 mg/g [29]. In this study, PEG solution was introduced during synthesis to leverage the preferential interaction between its hydrophilic hydroxyl groups and water (non-solvent). This mechanism reduces the miscibility driving force between DMF and water, slows the solidification rate of PAN molecular chains, and facilitates the formation of a more uniform pore network within the granular adsorbent, thereby significantly improving its performance [30]. Most current research efforts primarily focus on immobilizing KCuFC onto support materials to enable column-based adsorption, selecting appropriate desorption solvents, and studying the distribution coefficients of rubidium in the presence of interfering ions. However, few studies have addressed the concentrations of various ions in desorption solutions after the adsorbent interacts with complex brine systems. In the Qarhan Salt Lake, Rb^+^ is typically only about 7 mg/L. During the production of potassium salts, Rb^+^ can be enriched to 65 mg/L, at which point the solution contains 26,000 mg/L of K^+^, with a ratio of ρ(K^+^): ρ(Rb^+^) reaching 400. In actual production, a high adsorption rate is required to generate economic benefits. However, in brine with a high K^+^ content that has similar properties to Rb^+^, the adsorption effect of existing adsorbents will be greatly affected. Maintaining high adsorption efficiency results in reduced Rb^+^ loading on the adsorbent and increased K^+^ content, which introduces significant K^+^ contamination into the desorption solution [27]. Therefore, we draw on the water washing step in the extraction process and chromatography to enhance the elution step of the adsorbent to strengthen the separation and enrichment process [1,31]. In this work, a two-stage elution strategy was employed: 5 g/L NH_4_Cl solution was first applied to remove K^+^ impurities from the adsorbent through competitive ion exchange, leveraging the intermediate ionic radius of NH_4_^+^ (143 pm) between K^+^ (133 pm) and Rb^+^ (147 pm). Subsequently, 5 mol/L NH_4_Cl was utilized for Rb^+^ desorption to achieve high-purity recovery [32]. Through this method, when the overall adsorption process of Qarhan Salt Lake brine reaches an efficiency of 68%, the separation factor can reach 160, successfully achieving enrichment and separation of Rb^+^ in salt lake brine.

## 2. Results and Discussion

### 2.1. Material Characterization and Properties

**Figure 1:**
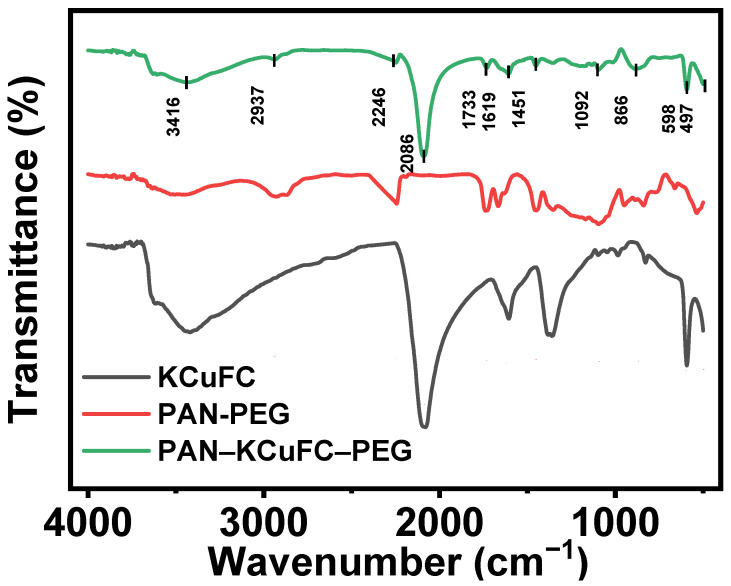
FT-IR spectrum of PAN-KCuFC-PEG, PAN-PEG, and KCuFC powder.

The Fourier infrared (FT-IR) spectra of the synthesized PAN-PEG, KCuFC, and porous PAN-KCuFC-PEG are displayed in Figure 1. Apparently, the FT-IR spectra of PAN-KCuFC-PEG exhibit the characteristic bands of PAN-PEG and KCuFC. The sharp and intense peaks observed at 2086 cm^−1^ are attributed to the stretching vibration of (C≡N), while the absorption band at 1619 cm^−1^ corresponds to the bending vibration of water molecules [33]. The absorption peaks (497 and 598 cm^−1^) located in the region of 450~600 cm^−1^ indicate the metal bonds C-Fe and C-Cu in the material, respectively [34], which proves that the KCuFC powder is successfully loaded into the PAN-KCuFC-PEG particles, and the overall structure is stable without significant changes.

**Figure 2:**
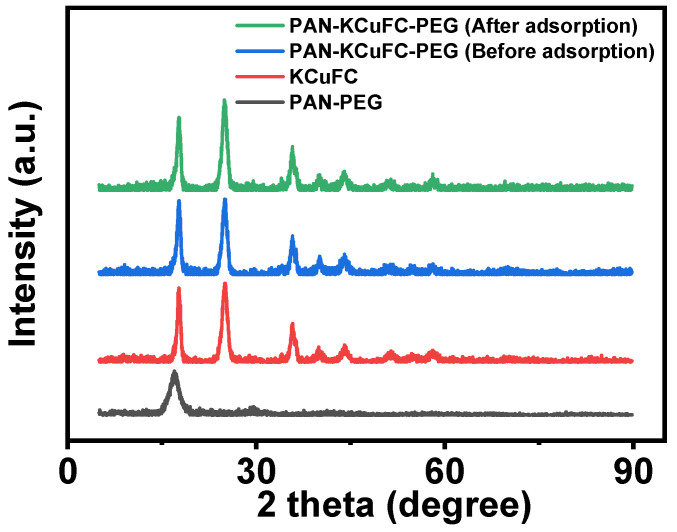
XRD pattern of the material.

The XRD patterns of PAN-PEG, KCuFC, and PAN-KCuFC-PEG before and after the adsorption of Rb^+^ are shown in Figure 2. As illustrated in Figure 2, the XRD pattern of KCuFC features distinct diffraction peaks with varying intensities at 2θ values of 17.8, 25.2, 36.0, 40.02, 44.3, 51.7, 54.0, and 58.2 (°), respectively. According to the diffraction plane of (200), (220), (400), (420), (424), (440), (600), and (620) in the copper ferrocyanide map (PDF#00-002-0376), the synthesized powder can be determined as a pure phase of KCuFC [35].

The relative peak strength of PAN-KCuFC-PEG on the (200) diffraction surface decreased significantly after adsorption of Rb^+^. The observed changes in peak intensity are attributed to the orientation of the crystal plane. KCuFC exhibits a crystal structure analogous to that of Prussian blue. The lattice parameters of ferrous potassium cyanide copper before the adsorption of rubidium ions are calculated as a = b = c = 10.027 Å and α = β = γ = 90°, and after the adsorption of rubidium ions, they are a = b = c = 10.043 Å and α = β = γ = 90°. After adsorbing rubidium ions, the crystal lattice expanded slightly, but the crystal structure did not change significantly.

**Figure 3:**
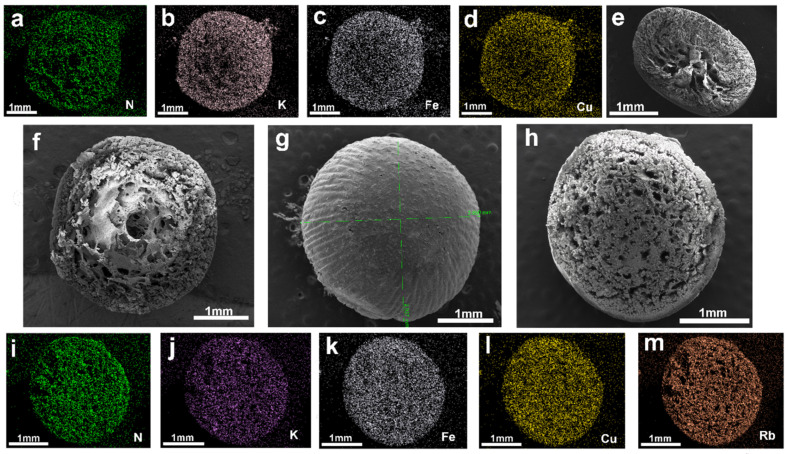
(**a**–**d**) Cross-sectional mapping images of PAN-KCuFC-PEG before adsorption; (**e**) cross-sectional view of the adsorbent without PEG addition; (**f**,**g**) cross-sectional and surface images of PAN-KCuFC-PEG before adsorption; (**h**–**m**) cross-sectional and mapping images of PAN-KCuFC-PEG after adsorption.

**Figure 4:**
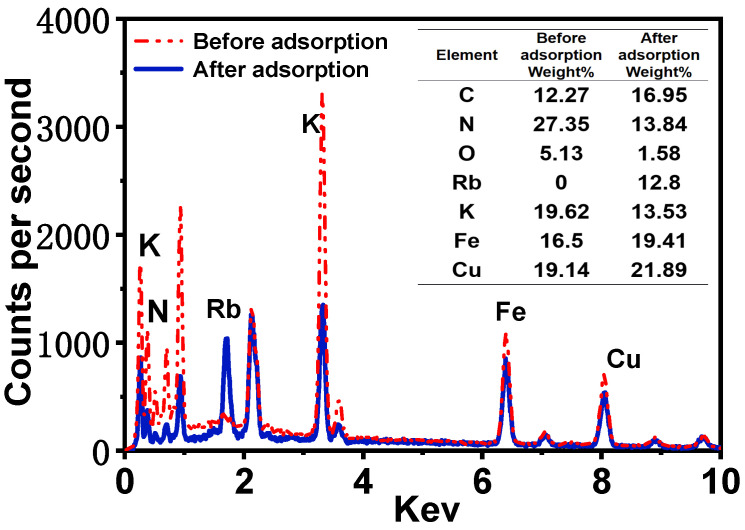
EDS spectra of PAN-KCuFC-PEG before and after Rb^+^ ion adsorption.

As illustrated in Figure 3g, scanning electron microscopy (SEM) images of the surface of PAN-KCuFC-PEG reveal that the spherical particles possess a structured, spherical morphology, with an approximate diameter of 2.9 mm. As illustrated in Figure 3f, the cross-section of the particle adsorbent is characterized by a porous and rough surface, indicative of its adsorbent properties. It is evident that the adsorbent exhibits a porous and rough appearance, and possesses a denser and more uniform pore structure in comparison to the particle adsorbent devoid of PEG, as depicted in Figure 3e. In addition, the SEM element mapping of K, N, Fe, and Cu on the PAN-KCuFC-PEG adsorbent (Figure 3a–d) quantitatively proves that all elements are distributed uniformly on the PAN-KCuFC-PEG substrate. The SEM mapping following the adsorption of Rb is displayed in Figure 3h–m. A substantial quantity of Rb elements is detected in PAN-KCuFC-PEG, thereby substantiating the adsorbent’s capacity to adsorb Rb. According to the EDX comparison before and after adsorption in Figure 4, it is evident that following the adsorption of Rb, a Rb peak emerges in the EDX spectrum, concurrent with a decline in the intensity of the K peak. This observation suggests that Rb has been adsorbed, while K has undergone an exchange.

The effect of PEG600 on the structure of PAN-KCuFC-PEG was investigated by BET characterization. Figure S2 and Table 1 show that the adsorption–desorption curves of the adsorbents before and after the addition of PEG are type IV isotherms. After the addition of PEG, the surface area of the adsorbents increases by 172% and the pore volume increases by 63%, which is conducive to the entry of brine [29]. This is consistent with the pore structure observed during SEM analysis. At the same time, the smaller aperture is more conducive to the coating of KCuFC powder.

### 2.2. Adsorption Performance of PAN-KCuFC-PEG

#### 2.2.1. Effect of Pore-Causing Agent on Adsorption Properties

To demonstrate the positive effect of the pore regulator PEG600 (MW = 600 g/mol, Fuchen Co., Ltd., Tianjin, China) on the pore structure, two different PAN-KCuFC-PEG spheres were prepared simultaneously: one without a pore-forming agent and the other using polyvinylpyrrolidone, commonly used for adjusting PAN films, as the pore-forming agent. The N_2_ adsorption–desorption isotherms are shown in Figures S1 and S2. Table 1 shows the adsorption properties of adsorbent beads with different pore-forming agents, as well as the pore volume distribution and BET surface area. The aperture of adsorbent spheres using PVP (Jinshan Chemical Reagent Co., Ltd., Chengdu, China) as a pore-forming agent becomes smaller and the pore volume becomes smaller, making it more difficult for brine to enter the particles. Therefore, porous PAN-KCuFC-PEG using PEG600 as a pore-forming agent has the best adsorption performance because it has the largest BET surface area and pore volume. The results show that PEG600 had a positive effect on the adsorption performance of PAN-KCuFC-PEG.

#### 2.2.2. Influence of pH on PAN-KCuFC-PEG

**Figure 5:**
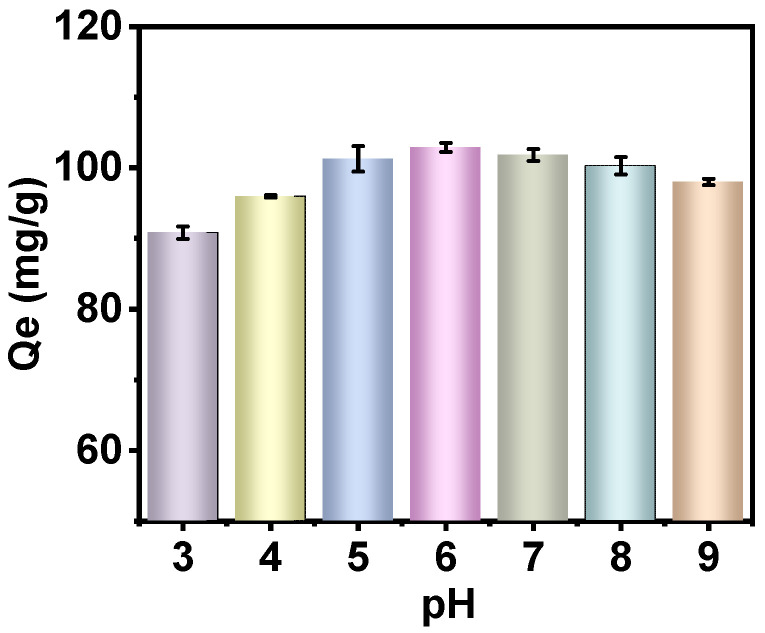
Influence of pH on Rb sorption by PAN-KCuFC-PEG (*C*_o_ = 60 mg Rb/L, sorbent dosage = 0.5 g/L).

As illustrated in Figure 5, PAN-KCuFC-PEG demonstrated a notable capacity for adsorbing Rb^+^ across a broad pH range. Notably, the adsorption capacity exhibited an increase in conjunction with an increase in pH from 3 to 6. However, under acidic conditions, a substantial amount of H^+^ and Rb^+^ competed for adsorption, thereby leading to a reduced adsorption capacity [21]. In the context of solution pH > 7, there was a slight decrease in adsorption capacity, yet the system demonstrated enhanced stability. As the pH level rises, the concentration of H^+^ ions in the solution declines, while the concentration of OH^−^ ions increases. This results in a strong force between iron ions and OH^−^ ions, leading to the fracture of the Fe-CN bond in KCuFC and the destruction of the material’s structure. Consequently, this reduces the material’s adsorption properties [36]. Concurrently, the rise in Na^+^ concentration within the same main group as Rb^+^ is predicted to compete with Rb^+^ for adsorption, thereby further diminishing the adsorption of Rb^+^ [8].

#### 2.2.3. Effect of Adsorption Time on PAN-KCuFC-PEG Adsorption of Rb^+^ and Its Kinetic Model Fitting

**Figure 6:**
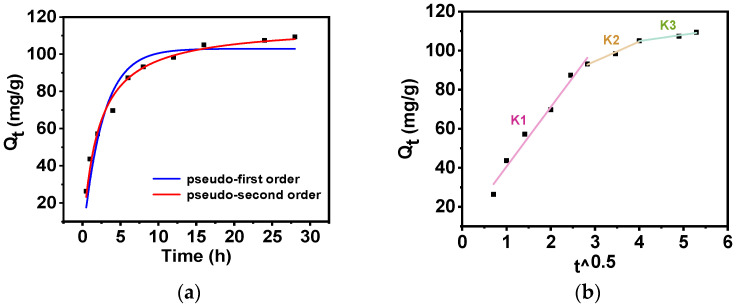
(**a**) Kinetics model fit (—PFO; —PSO) and (**b**) intraparticle diffusion model (*C*_o_= 60 mg Rb/L, sorbent dosage = 0.5 g/L).

In order to study the adsorption behavior of Rb^+^ on PAN-KCuFC-PEG, the effect of adsorption time on the ability of PAN-KCuFC-PEG to adsorb Rb^+^ was tested, and the pseudo-first-order model (PFO) and pseudo-second-order model (PSO) kinetic adsorption models were established, as shown in Figure 6a and Table 2. The PSO model has a high correlation coefficient (*R*^2^ = 0.98), which indicates that the model can effectively characterize the adsorption data. It shows that chemisorption occurs in this process [37]. Surface chemisorption and the number of active sites on the surface of the material are the main factors affecting the adsorption process [28].

According to the intraparticle diffusion model, the adsorption process of Rb^+^ on PAN-KCuFC-PEG is divided into three stages, as illustrated in Figure 6b and Table 3. This indicates that multiple processes control the adsorption process. The slope of the linear fit in the first stage (0–8 h) is relatively steep, which implies that there are abundant adsorption sites and a high adsorption rate. Nevertheless, the linear fit does not pass through the origin, suggesting the presence of intraparticle diffusion and external mass transfer. The enlarged pore volume and surface area of PAN-KCuFC-PEG enable favorable adsorption during this period. In the subsequent stage (8–16 h), a decline in the slope of the fit line signifies a reduction in the adsorption sites, consequently leading to a decrease in the adsorption rate. At the third stage (from 16 to 28 h), upon the decrease in the solution concentration, the mass transfer force decreases, which indicates that the adsorption process ultimately attains its equilibrium state. This finding suggests that the adsorption process is not solely constrained by intraparticle diffusion.

#### 2.2.4. Rb Adsorption Isotherms of PAN-KCuFC-PEG

**Figure 7:**
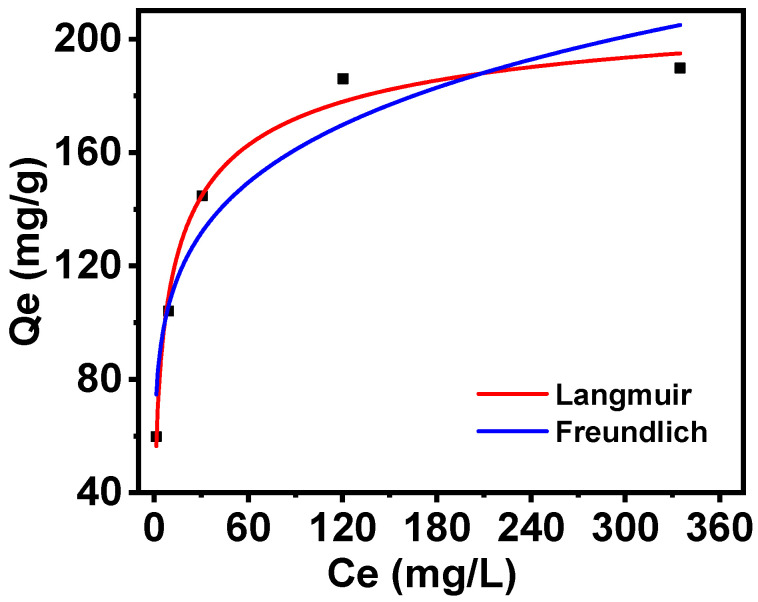
Isotherm nonlinear fitting by (—Langmuir) model and (—Freundlich) model for different initial concentrations of Rb^+^ adsorption on PAN-KCuFC-PEG.

The Langmuir and Freundlich isotherm models were employed to fit the adsorption isotherm of PAN-KCuFC-PEG, the results of which are shown in Figure 7. The Langmuir model of Rb^+^ adsorption exhibited the greatest fit for the adsorbent data, with a higher correlation coefficient (*R*^2^ = 0.98), while the Freundlich curve demonstrated greater dispersion. At a temperature of 25 °C, the maximum adsorption capacity calculated by the Langmuir model (223 mg/g) closely corresponds to the experimental value (190 mg/g). Consequently, the adsorption behavior of PAN-KCuFC-PEG on Rb^+^ is characterized as single-layer adsorption, with its effective adsorption sites distributed evenly.

#### 2.2.5. PAN-KCuFC-PEG Performance in the Presence of Salt Lake Brine Ions

The most challenging aspect of extracting Rb^+^ from Qarhan Salt Lake brine is the presence of a substantial number of alkali metal ions and bivalent cations within the brine, which possess analogous properties and exert a pronounced influence on the adsorption of Rb^+^. To investigate the impact of Li^+^, Mg^2+^, Na^+^, Ca^2+^, and K^+^ on Rb^+^ extraction, we have developed five groups of solutions with varying mass concentrations of M/Rb (M = Li, Mg, Na, Ca, K) at ratios of 3:1, 1100:1, 230:1, 10:1, and 400:1, with a constant *C*_Rb_ of 60 mg/L.

**Figure 8:**
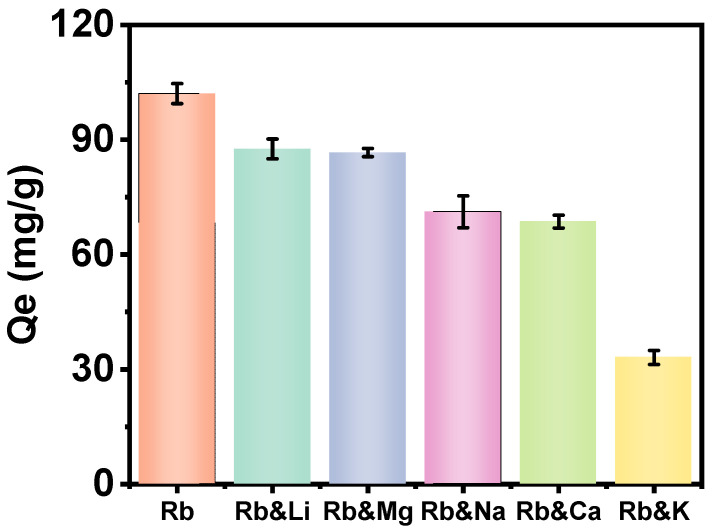
Interference of metal ions on adsorption capacity for Rb^+^.

As illustrated in Figure 8, the adsorption capacity of Rb^+^ on PAN-KCuFC-PEG was examined. We found that potassium ions in the salt lake have the greatest impact on the adsorption of rubidium ions, reducing the adsorption capacity of Rb^+^ by 69%. As shown in Table 4, this may be due to the similar ionic radii and hydration energies between potassium ions and rubidium ions, which allow K^+^ ions to compete with Rb^+^ more effectively than other metal ions [38]. The Lewis acid–base theory and Pearson’s soft–hard acid–base concept can also explain these experimental phenomena [39]. As the atomic numbers of sodium, magnesium, potassium, calcium, and rubidium increase, the attraction of the atomic nucleus to the outermost electrons gradually weakens. Therefore, the Rb^+^ ion is classified as a Lewis acid, and its properties are closer to those of a soft acid compared to other interfering ions [40]. Because the Rb^+^ may more readily combine with soft Lewis bases, such as the cyanide group (-CN), the PAN-KCuFC-PEG particulate adsorbent exhibits the highest selective adsorption capacity for rubidium ions and is suitable for Rb^+^ extraction under high-salinity conditions, such as those in the brine of Qarhan Salt Lake.

#### 2.2.6. Comparison of Various Adsorbents for Rb^+^ Adsorption

A performance comparison of the PAN-KCuFC-PEG adsorbent in this study with commonly reported adsorbents for rubidium and cesium (detailed results in Table 5) reveals that its adsorption capacity is comparable to or even superior to those of various Prussian blue analogue-based composite materials. Furthermore, PAN-KCuFC-PEG demonstrates outstanding adsorption performance when compared to conventional ammonium molybdophosphate-based materials and other adsorbent materials.

### 2.3. PAN-KCuFC-PEG Fixed-Bed Column

#### 2.3.1. Sorption Capacity

**Figure 9:**
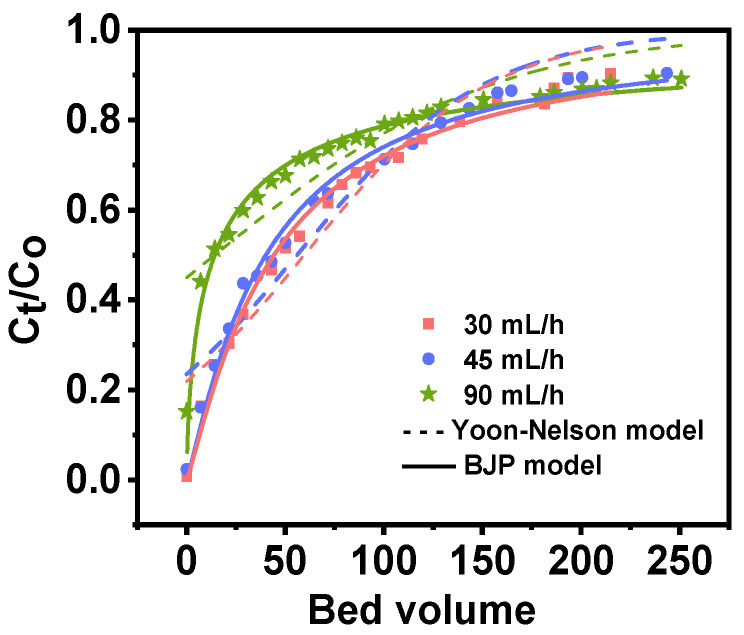
The breakthrough curves of rubidium adsorption in a fixed-bed column of PAN-KCuFC-PEG under different flow rates.

Following preliminary separation, the concentration of major ions in Qarhan Salt Lake is presented in Table 6. Rb^+^ is enriched to 65 mg/L; however, the content of interfering ions remains thousands of times that of Rb^+^. As illustrated in Figure 9, the breakthrough curve was obtained from 2 g of PAN-KCuFC-PEG treated with 5 mol/L ammonium chloride at flow rates of 90, 45, and 30 mL/h following the initial separation of the Qarhan Salt Lake brine. The corresponding empty bed contact time, defined as bed volume to volume flow rates of 8.37, 16.75, and 25.12 min, respectively, was considered. The bed height was maintained at 16 cm, and the temperature was fixed at 25 °C. It can be deduced that the shape of the breakthrough curve remains similar under varying flow rates. As the flow rate increases, the breakthrough and saturation times decrease.

When feeding brine of identical bed volume into the adsorption column at flow rates of 30, 45, and 90 mL/h, the effluent concentration increases and the adsorption capacity of the adsorbent diminishes with higher flow rates. This is related to the fact that the increase in flow rate shortens the contact time between Rb^+^ and the adsorbent, resulting in the weakening of the interaction between Rb^+^ and the adsorbent [36]. On the other hand, the high flow rate gives less time for Rb^+^ to diffuse within the adsorbent particles, accelerating breakthrough and saturation. We used Yoon–Nelson and BJP models to fit the adsorption process. In the BJP model (*R*^2^ = 0.98), the smaller the *K*_BJP_ value, the longer the breakthrough time [44]. When the flow rate is 30 mL/h, the calculated saturation adsorption capacity (30.62 mg/g) in the column increases compared with the simulated solution containing only the same potassium ion content. This is because the feed liquid concentration in the column adsorption is always at a higher level, and the driving force is enhanced. To ensure that the overall yield of Rb^+^ in the adsorption–desorption process exceeds 65%, we selected a breakthrough point of 70% to carry out the subsequent experiment, and calculated the adsorption capacity of the breakthrough point in the column at a flow rate of 30 mL/h (7.19 mg/g).

#### 2.3.2. Washed Columns

**Figure 10:**
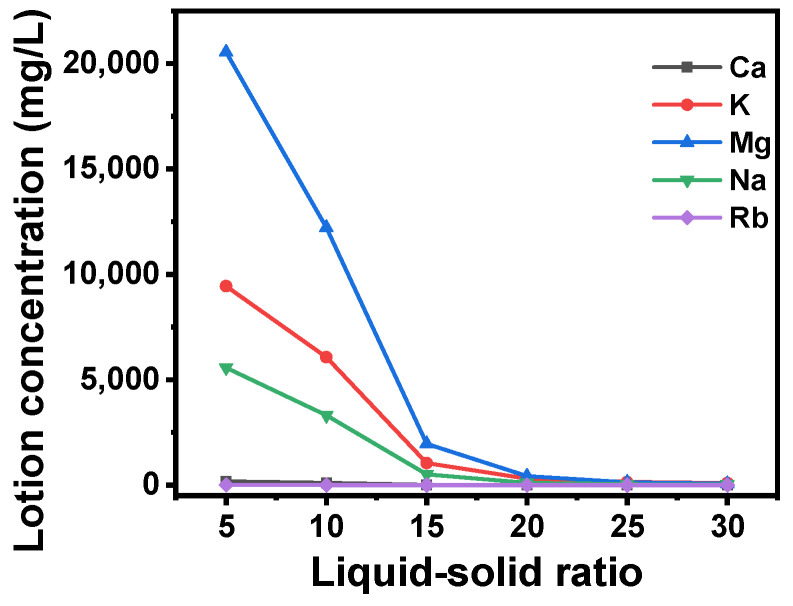
Influence of variations in the liquid–solid ratio of deionized water washing solution on the concentrations of various ions at the washing solution outlet.

Subsequently, the column post-adsorption underwent rinsing with deionized water. This process was carried out to eliminate the solution entrapped within the adsorbent particles. The ion concentrations at the outlet of the washing solution are presented in Figure 10. It was observed that the adsorbed brine contained a significant amount of Mg^2+^, Na^+^, and K^+^, resulting in elevated ion concentrations in the lotion at the outset. However, as the liquid–solid ratio of the lotion increased, the ion concentrations decreased. The ion concentrations in the lotion were found to be less than 100 mg/L. When the solid–liquid ratio reaches 20, the ion concentrations of Mg^2+^, Na^+^, and K^+^ at the exit are 429.4, 112.7, 321.3 mg/L, and the ion concentrations of Ca^2+^ are almost 0.

#### 2.3.3. Wash with Low Concentration of NH_4_Cl

**Figure 11:**
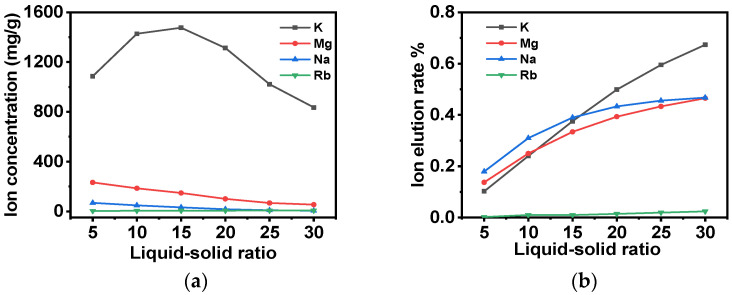
(**a**) The effect of changes in the liquid–solid ratio of low-concentration ammonium chloride washing solution on ion concentrations at the washing solution outlet; (**b**) the influence of changes in the liquid–solid ratio of low-concentration ammonium chloride washing solution on the removal rates of different ions from the adsorbent.

Subsequently, after the washing of the adsorbent with deionized water, the adsorbent is to be washed with a 5 g/L NH_4_Cl eluent. The outlet concentration of the washing solution and the elution rate of each ion in the adsorbent are shown in Figure 11a and Figure 11b, respectively. It is evident from these data that the removal rates of K^+^, Mg^2+^, Na^+^, and Rb^+^ increase in proportion to the gradual increase in the liquid–solid ratio of the lotion. When the liquid–solid ratio is 30, the concentration of Rb^+^ reaches 7 mg/L, which is close to 10% of the concentration of the original solution, and 67% of K^+^ can be eluted from the adsorbent, although the concentration of K^+^ at the outlet of the effluent is still 835 mg/L. However, if the liquid volume continues to increase, the rubidium yield is found to be inadequate. Therefore, a liquid–solid ratio of 30 is selected to achieve a high separation effect and ensure a high yield.

#### 2.3.4. Desorption Experiments

**Figure 12:**
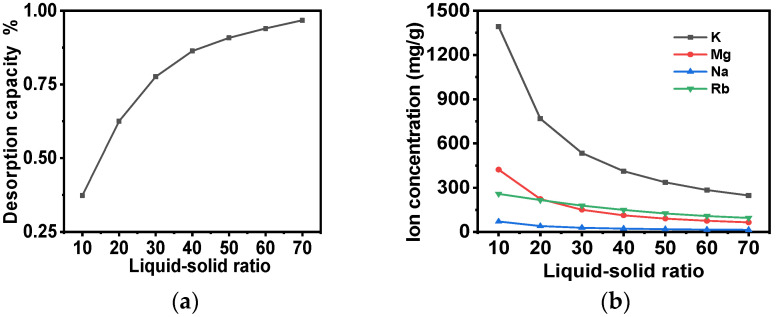
(**a**) The influence of the variation in the liquid-to-solid ratio of ammonium chloride desorption solution at 60 °C on the desorption of the adsorbent; (**b**) the impact of the alteration in the concentration of each ion in the desorption solution during desorption at 60 °C.

Following the elution of the adsorbent with a low concentration of NH_4_Cl lotion, the 5 mol/L NH_4_Cl desorption liquid was introduced into the column, and the adsorbent was desorbed at 60 °C. When a high-concentration ammonium chloride solution undergoes desorption, the mass transfer driving force during desorption is significantly enhanced, drastically reducing the amount of solvent required. This reduction in solvent usage directly elevates the rubidium concentration in the desorbed solution, thereby achieving effective enrichment of Rb through desorption. The impact of the variation in the liquid-to-solid ratio of the ammonium chloride desorption liquid on the desorption rate and the alteration in the concentration of ions in the desorption liquid at 60 °C is illustrated in Figure 12. It is evident that as the liquid–solid ratio increases at 60 °C, the resolved rubidium continues to escalate. Furthermore, the initial K^+^ content of the effluent reaches 1400 mg/L. However, when the liquid–solid ratio reaches 20, the K^+^ content of the effluent decreases to 144 mg/L, and the average K^+^ content of the desorption solution decreases from 1392.14 mg/L to 768 mg/L. Furthermore, when the liquid–solid ratio is 70, the K^+^: Rb^+^ ratio in the desorption solution decreases from 400:1 to 2.5:1. In comparison with the initial solution containing K^+^ (26,000 mg/L) and Rb^+^ (65 mg/L), the separation factor of the entire adsorption process exhibits a substantial increase to 160. Concurrently, the desorption rate and the overall yield of the adsorption process demonstrate notable improvements, reaching 96.6% and 67.6%, respectively. This outcome signifies the effective realization of the separation and enrichment of Rb^+^ from a saline solution with high salt content.

## 3. Materials and Methods

### 3.1. Materials

Copper(II) nitrate trihydrate (Cu(NO_3_)_2_·3H_2_O) was procured from Shanghai Aladdin Biochemical Science and Technology Co., Ltd. (Shanghai, China); polyacrylonitrile (PAN, average MW = 50,000 g/mol) was obtained from Shanghai Taitan Science and Technology Co., Ltd. (Shanghai, China). Polyethylene glycol (PEG600, MW = 600 g/mol) was acquired from Fuchen (Tianjin, China) Chemical Reagent Co., Ltd. N,N-dimethylformamide (DMF) was purchased from Chengdu Kelong Chemical Co., Ltd. (Chengdu, China). Potassium ferricyanide trihydrate (K_4_Fe(CN)_6_·3H_2_O), KCl, NaCl, NH_4_Cl and polyvinylpyrrolidone (PVP K30) were procured from Chengdu Jinshan Chemical Reagent Co., Ltd. (Chengdu, China). It is noteworthy that all reagents were of analytical grade and could be used without further purification.

### 3.2. Synthesis of PAN-KCuFC-PEG

The synthesis of KCuFC powder is outlined as follows: A 50 mL solution of 0.5 mol/L K_4_(Fe(CN)_6_)·3H_2_O is gradually added to a 50 mL solution of 0.5 mol/L Cu(NO_3_)_2_·3H_2_O. The resultant mixture is stirred at room temperature for 1.5 h, then washed 3 times with deionized water, subjected to centrifugation, and dried at 55 °C for 24 h. Subsequently, the dried product is ground and sifted through a 200-mesh sieve for subsequent use.

**Figure 13:**
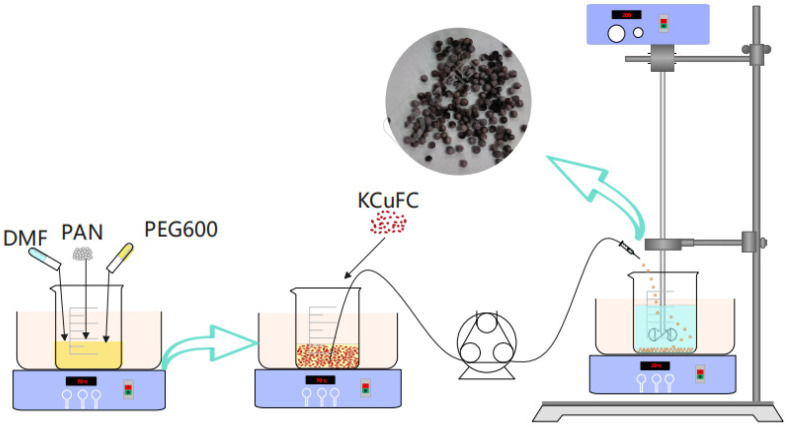
The preparation process of PAN-KCuFC-PEG spheres.

We conducted single-factor experiments in the simulated brine using PAN-KCuFC-PEG, systematically optimizing the ratio of KCuFC powder in the particulate adsorbent, the mass ratio of the particulate adsorbent to the solvent DMF, the synthesis temperature, and the mass ratio of PEG to DMF. The experimental results are shown in Figures S3–S6 of the Supporting Material. A total of 0.5 g of PAN was dissolved in 6 g of N,N-dimethylformamide, and 0.25 g of PEG600 pore conditioner was subsequently added. The mixture was stirred for 1 h at 70 °C to ensure complete dissolution and homogeneity. Subsequently, 1 g of KCuFC powder was incorporated and the mixture was stirred for an additional 1 h to ensure the attainment of a homogeneous solution. The resultant mixture was then transferred into a beaker containing deionized water using a syringe pump fitted with a needle. The mixture was thoroughly rinsed with deionized water multiple times to ensure the removal of the PEG600 and PAN-KCuFC-PEG. Subsequently, the mixture was oven-dried at a temperature of 45 °C for a duration of 24 h. The preparation of the PAN-KCuFC-PEG beads was carried out in accordance with the steps depicted in Figure 13.

### 3.3. Characterisation of PAN-KCuFC-PEG

The microstructure and morphology of the prepared products were investigated by scanning electron microscopy (Inspect F50, Thermo Fisher Scientific, Cambridge, MA, USA). The surface and cross-sectional morphology and elemental content of the products before and after the adsorption of Rb were evaluated by energy dispersive X-ray spectroscopy (Inspect F50, Thermo Fisher Scientific, Cambridge, MA, USA). The functional groups of all samples were determined by Fourier transform infrared spectroscopy (IRTracer 100, Shimadzu Corporation, Kyoto, Japan) in the wavenumber range of 400–4000 cm^−1^. X-ray diffraction (X’Pert3 Powder, Malvern Panalytical, Amsterdam, NL, USA) in the scanning range of 5–70° was used to collect information on the crystal structure of the adsorbents. Nitrogen adsorption/desorption isotherms (ASAP 2460, Micromeritics, San Jose, CA, USA) were used to analyze the pore properties of the adsorbent. The specific surface area was evaluated by the Brunauer–Emmett–Teller (BET) method, based on the nitrogen adsorption data.

### 3.4. Sorption Experiments

#### 3.4.1. Adsorption Kinetics

The experiment was conducted using 0.5 g/L PAN-KCuFC-PEG particulate adsorbent, which was placed in a glass flask. Subsequently, 100 mL of a solution with an Rb^+^ concentration of 60 mg/L was added. The adsorption experiments were conducted within a water bath shaker, equipped with a controlled water temperature of 25 ± 0.5 °C. Samples were collected at 0.5,1, 2, 4, 6, 8, 12, 16, 24, and 28 h intervals using a syringe with a 0.22 μm pore-size filter. The residual Rb^+^ concentration in the solution was analyzed by inductively coupled plasma emission spectrometry (ICP-6300, Thermo Fisher Scientific, Cambridge, MA, USA). The equilibrium adsorption (*Q*_t_) of the adsorbent at each time interval was calculated using Equation (1) based on the Rb^+^ concentration.

(1) $$Q_{t}=\frac{(C_{0}-C_{t})V}{m}$$

where *C*_t_ and *C*_0_ represent the residual and initial concentrations (in milligrams per liter), respectively. The quantity *Q*_t_ denotes the amount of Rb^+^ that has been adsorbed at a given time (in milligrams per gram). The variables *V* and *m* correspond to the volume (in liters) and mass (in grams) of the adsorbent present in each sample, respectively [45].

Detailed kinetic models are described in the Supplementary Materials.

#### 3.4.2. Adsorption Isotherms

The 0.5 g/L PAN-KCuFC-PEG particle adsorbent was added into 100 mL (30–400 mg/L) Rb^+^ solution with different concentrations, and the temperature was maintained at 25 ± 0.5 °C for 24 h under shock adsorption. Prior to analyzing the remaining Rb^+^ concentration in the solution, samples were collected with a syringe fitted with a filter with a pore size of 0.22 μm. The remaining Rb^+^ concentration in the solution was then analyzed by ICP-6300.

The Langmuir and Freundlich isotherm models were obtained by using nonlinear regression equations to fit the experimental data of Rb^+^ adsorption.

A detailed adsorption isotherm model is described in the Supplementary Material [46].

### 3.5. Column Experiments

In our investigation of the existing production processes at Qarhan Salt Lake, we observed that Rb could be enriched to 65 mg/L during the potassium production process, with the ion concentrations in the solution provided in Table 5. This brine was subsequently utilized for column adsorption experiments. Dynamic adsorption tests were carried out in a fixed bed column (1 cm inner diameter, 20 cm length, 16 cm bed height) filled with 2 g of PAN-KCuFC-PEG, as illustrated in Figure S7. Initially, 5 mol/L NH_4_Cl solution was injected into the adsorption column to replace the original K^+^ of the adsorbent. Thereafter, the brine was pumped through the column at flow rates of 30, 45, and 90 mL/h for the adsorption experiments. Effluent from the column outlet was collected at regular intervals, and the concentrations of Rb^+^, K^+^, Na^+^, Ca^2+^ and Mg^2+^ were determined using inductively coupled plasma mass spectrometry (ICP). The breakthrough curve was typically expressed as the inlet Rb concentration (*C*_0_), outlet Rb concentration (*C*_t_), or normalized concentration. The normalized concentration was defined as the ratio of Rb outlet concentration to Rb inlet concentration (*C*_t_/*C*_0_). The breakthrough time was defined as the time when the Rb^+^ concentration of outlet water reached 30% of the inlet water concentration. The saturation time was defined as the time when the Rb^+^ concentration at the outlet reached 90%. The Yoon–Nelson model and the BJP model of Equations (2) and (3) were employed to fit the experimental data.

(2) $$\mathrm{Yoon}\text{-}\mathrm{Nelson}:\;\frac{C_{t}}{C_{0}}=\frac{\exp(\frac{K_{YN}\times n\times V}{Q}-K_{YN}\times \tau )}{1+\exp(\frac{K_{YN}\times n\times V}{Q}-K_{YN}\times \tau )}$$

*K*_YN_ represents the rate constant of the Yoon–Nelson model, (min^−1^); *τ* is the time when *C*_t_/*C*_0_ equals 0.5; n is the number of bed volumes; *V* represents the volume of a unit bed layer, (mL); *Q* represents the flow rate of the brine, (mL/h).

(3) $$\mathrm{BJP}:\;\frac{C_{t}}{C_{0}}=\frac{1}{1+{\left(K_{BJP}\times \frac{n\times V}{Q}\right)}^{P}}$$

*p* is a dimensionless constant; *K_BJP_* is the rate constant of BJP model, (min^−1^); n is the number of bed volumes; *V* represents the volume of a unit bed layer, (mL); *Q* represents the flow rate of the brine, (mL/h).

The following equation can be used to calculate the adsorption capacity:

(4) $$q_{t}=\frac{Q}{m}\int_{0}^{t}(C_{0}-C_{t})dt$$

In this equation, *q_t_* denotes the adsorption capacity at equilibrium (mg/g), *Q* represents the flow rate (mL/h), and *m* signifies the mass of the adsorbent (g).

Subsequent to the adsorption process, deionized water was introduced into the column, and the concentration of Rb^+^, K^+^, Na^+^, Ca^2+^ and Mg^2+^ at the effluent outlet was measured by an ICP analyzer. This procedure was intended to remove the ions entrapped within the adsorbent particles. Following the initial washing procedure, the adsorption column was subjected to a secondary washing treatment using 0.5 g/L NH_4_Cl through chromatography. This process was specifically designed to remove the K^+^ ions that had become bound to the adsorbent during the adsorption process. The concentration of Rb^+^, K^+^, Na^+^, Ca^2+^ and Mg^2+^ at the outlet of the effluent was measured by ICP analysis. The 5 mol/L NH_4_Cl solution was introduced into the adsorption column at a temperature of 60 °C for desorption experiments. The concentrations of Rb^+^, K^+^, Na^+^, Ca^2+^ and Mg^2+^ at the effluent outlet were measured using an ICP analyzer, and Equation (5) was employed to calculate the separation factors of K and Rb during the entire adsorption and desorption process.

(5) αKRb=yKxkyRbxRb

## 4. Conclusions

In summary, a highly stable PAN-KCuFC-PEG microsphere porous composite was prepared for the extraction of rubidium from salt lake brine. The results show that the addition of PEG increases the surface area and pore volume of PAN-KCuFC-PEG microspheres, which is beneficial for mass transfer and enhances the adsorption performance reaching 190 mg/g. The fixed-bed column loaded with PAN-KCuFC-PEG achieved a saturation adsorption capacity of 30.62 mg/g for rubidium from actual salt lake brine, with a breakthrough adsorption capacity of 7.19 mg/g. When the adsorption rate of Qarhan Salt Lake brine reaches 70% and the desorption rate reaches 96.6%, the total yield of the adsorption process reaches 68%, and the overall separation coefficient is 160, indicating that it has a good separation effect on salt lake brine. The efficacy of PAN-KCuFC-PEG in facilitating the separation and enrichment of Qarhan Salt Lake brine is evident, thereby paving the way for subsequent separation and purification processes.

## Figures and Tables

**Table 1:** Pore size, surface area, and pore volume of PAN-KCuFC-PVP, PAN-KCuFC-PEG and KCuFC-PAN.

Sample	Pore Volume	BET Surface Area	Average Pore Diameter	*Q*e
	(cm^3^/g)	(m^2^/g)	(nm)	(mg/g)
KCuFC-PAN	0.0205	5.30	26.93	54.61
KCuFC-PAN-PEG	0.0335	14.46	11.99	62.17
KCuFC-PAN-PVP	0.0162	5.611	13.31	50.84

Adsorption conditions: 55 mg/L Rb^+^ solution, 0.6 g/L adsorbent dose, pH = 7, 2 h and 25 °C.

**Table 2:** Fitting of PFO and PSO to the adsorption kinetic data of Rb^+^ on PAN-KCuFC-PEG.

Pseudo-First-Order Model			Pseudo-Second-Order Model		
*Q*_t_ (mg/g)	*K*_1_ (/h)	*R* ^2^	*Q*_t_ (mg/g)	*K*_2_ (/h)	*R* ^2^
102	0.37	0.94	115	0.004	0.98

**Table 3:** Fitting of intraparticle diffusion model to the adsorption kinetic data of Rb^+^ on PAN-KCuFC-PEG.

	Parameter	PAN-KCuFC-PEG
K1	*K* (mg/g/h^0.5^)	30.47
	*C* (mg/g)	10.10
	*R* ^2^	0.97
K2	*K* (mg/g/h^0.5^)	10.05
	*C* (mg/g)	64.38
	*R* ^2^	0.98
K3	*K* (mg/g/h^0.5^)	3.24
	C (mg/g)	91.95
	*R* ^2^	0.97

**Table 4:** Hydrated and unhydrated ionic radii of alkali metals, Mg and Ca.

Metal	Hydrated Ionic Radius, Å [36]	Unhydrated Ionic Radius, Å [35]
Rb	2.26–2.28	1.68
K	2.32–3.31	1.48
Ca	4.1–4.13	1.01
Na	2.76–3.60	0.95
Mg	4.28	0.65
Li	3.4–4.7	0.6

**Table 5:** The adsorption performance of various adsorbents.

Entry	Materials	Adsorption Capacity (mg/g)		Ref.
		Rb	Cs	
1	PH-@MIL-101	73.1		[41]
2	AMP-SiO_2_	24.67		[14]
3	PAN-KCuFC	104.5		[27]
4	CMC–KCuFC		60.8	[28]
5	PVC-SSbPP	81.3	95.23	[12]
6	AMP2.4/PSf	62.89		[39]
7	CMC–KCuFC-PEG		149.8	[29]
8	Fe_3_O_4_@ZIF-8@AMP	53	78.276	[42]
9	KCuFC/SPSG	165.4		[43]
10	PAN-KCuFC-PEG	190		This work

**Table 6:** Composition of after treatment Qinghai brine.

Parameter	Value
pH	6.4
Inorganic cations (mg/L):	
Ca	540
Mg	72,900
Li	175
Na	15,000
K	26,000
Rb	65

## Data Availability

The data that support the findings of this study are available upon request.

## Supplementary Materials

The following supporting information can be downloaded at https://www.mdpi.com/article/10.3390/molecules30061273/s1: Figure S1: Nitrogen adsorption–desorption isotherms for PAN-KCuFC and PAN-KCuFC-PEG. Figure S2: Nitrogen adsorption–desorption isotherms for PAN-KCuFC-PVP. Figure S3: Effect of KCuFC powder content in granular adsorbent on adsorption performance. Figure S4: The effect of the mass ratio of DMF to granular adsorbent on adsorption performance. Figure S5: The effect of synthesis temperature on the adsorption performance of granular adsorbent. Figure S6: The effect of the mass ratio of PEG to DMF on the adsorption capacity. Figure S7: Schematic diagram of adsorption column adsorption. Table S1: Simulated Brine Composition.
