# Supplementary material for: Efficient Separation and Enrichment of Rubidium in Salt Lake Brine Using High-Performance PAN-KCuFC-PEG Adsorption Composite
Source: Molecules. 2025 Mar 12;30(6):1273. doi: 10.3390/molecules30061273 (PMC11944588; doi:10.3390/molecules30061273)
Supplement: Supplementary file 1 [file molecules-30-01273-s001.zip › molecules-3497873-supplementary.pdf]

Supporting Information for

Efficient Separation and Enrichment of Rubidium in Salt Lake Brine Using High - Performance PAN-KCuFC-PEG Adsorption Composite

Linhong Wu[1], Kun Zhou[1,*], Yuchen Zhen[1], Ying Zeng[1], Guangyong Zeng[1], Ziyi Chen[1] and Yang Peng[1]

1. College of Materials and Chemistry & Chemical Engineering, Chengdu University of Technology, Chengdu, 610059, P.R. China.

* Correspondence: zhoukun2013@cdut.edu.cn (K.Z.)

Materials and methods

2.2.3. Adsorption kinetics

To characterize the adsorption process, we fitted the kinetic adsorption results to pseudo-first-order (PFO) and pseudo-second-order (PSO) models using nonlinear regression and the least-squares approach. The following equations represent the nonlinear forms of the PFO and PSO kinetic models, respectively:

Pseudo-first order: $Q_t = Q_{e1}(1 - e^{-K_1 t})$ (1)

Pseudo-second order: $Q_t = Q_{e_2}{}^2 k_2 t / (1 + Q_{e_2} k_2 t)$ (2)

2.4.2. *Adsorption isotherms*

Langmuir and Freundlich models

Langmuir isotherm: $Q_e = (K_L C_e Q_{\max}) / (1 + K_L C_e)$ (3)

Freundlich isotherm: $Q_e = K_f C_e^{1/n}$ (4)

where C_e is the equilibrium Rb concentration in solution phase (mg/L), Q_e denotes the Rb sorption capacity (mg/g) at equilibrium, Q_{max} is the maximum adsorption at monolayer (mg/g), and K_L is the Langmuir isotherm constant; K_f and n are the Freundlich constants.

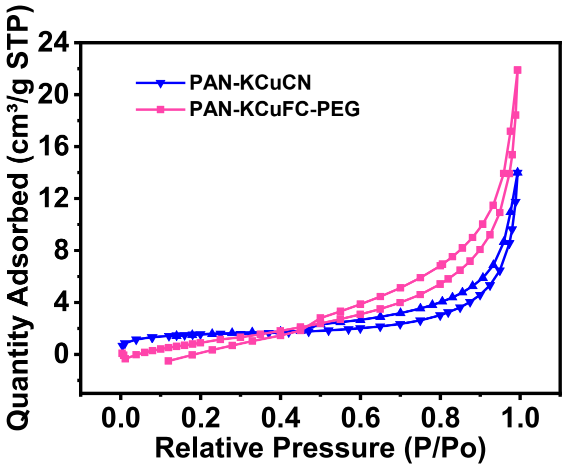

Figure. S1 Nitrogen adsorption–desorption isotherms for PAN-KCuFC and PAN-KCuFC-PEG

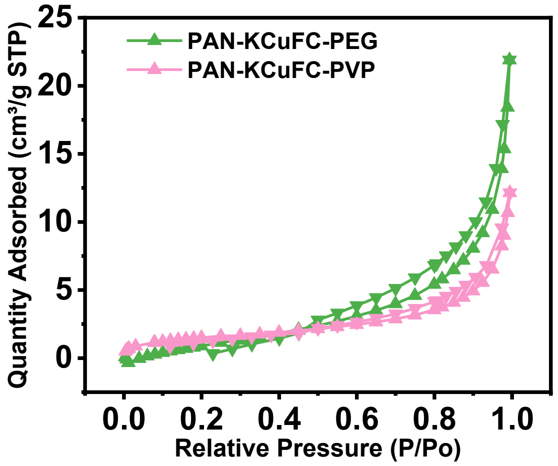

Figure. S2 Nitrogen adsorption–desorption isotherms for PAN-KCuFC-PVP SEM image

3.2. Synthesis of PAN-KCuFC-PEG

We conducted single-factor experiments in the simulated brine using PAN-KCuFC-PEG, systematically optimizing the ratio of KCuFC powder in the particulate adsorbent, the mass ratio of the particulate adsorbent to the solvent DMF, the synthesis temperature, and the mass ratio of PEG to DMF.

1. In a water bath at 50℃, 0.5 g of PAN was dissolved in 5 g of DMF and stirred to obtain a uniform solution. Different amounts of KCuFC powder (1, 2, 3, 4, and 5 g) were added respectively, and the mixture was stirred for an additional hour to obtain a mixed slurry. Then, the granular adsorbent was prepared, ensuring that the proportion of KCuFC in the composite PAN-KCuFC granular adsorbent was 0.67, 0.8, 0.86, 0.89, and 0.91. The simulated brine composition is shown in Table S1. 1 g of the synthesized PAN-KCuFC-PEG was added to 500 mL of simulated rubidium chloride (RbCl) solution, and the mixture was shaken for 4 hours to perform adsorption. After adsorption, the supernatant was collected to analyze the Rb^+ concentration, and the adsorption efficiency was evaluated as shown in Figure S3.

Table S1: Simulated Brine Composition

	Na	K	Rb

Concentration (mg/L)	12	12	0.06

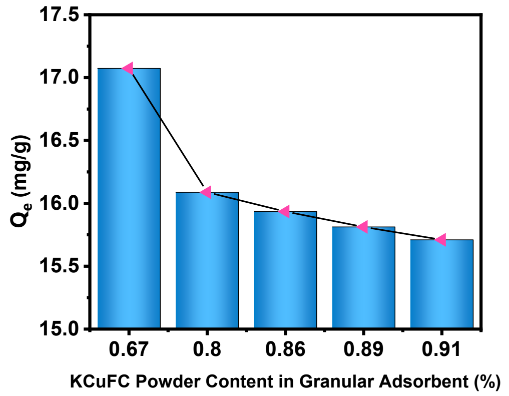

Figure S3. Effect of KCuFC powder content in granular adsorbent on adsorption performance.

2. In a water bath at 50℃, 0.5 g of PAN was dissolved in 3, 4, 5, 6, and 6.5 g of DMF solution respectively, and stirred to obtain a uniform solution. Then 1 g of KCuFC powder was added and the mixture was stirred for another hour to obtain the mixed slurry. The granular adsorbent was prepared in a water-solidified bath, ensuring the mass ratio of DMF to granular adsorbent in the mixed slurry was 2, 2.67, 3.33, 4, and 4.33. 1 g of the synthesized PAN-KCuFC-PEG was added to 500 mL of simulated rubidium chloride (RbCl) solution, and the mixture was shaken for 4 hours to perform adsorption. After adsorption, the supernatant was collected to analyze the Rb^+ concentration, and the adsorption efficiency was evaluated as shown in Figure S4.

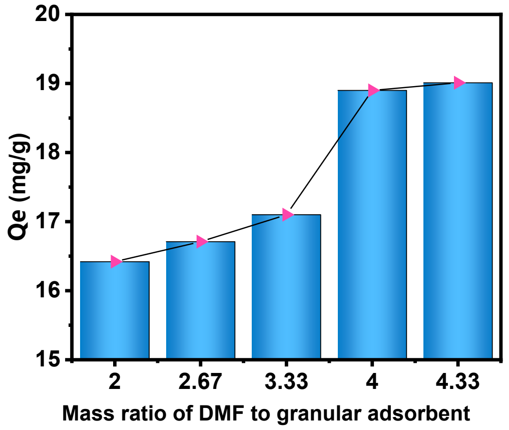

Figure S4. The effect of the mass ratio of DMF to granular adsorbent on adsorption performance.

3. In water baths at temperatures of 30, 40, 50, 60, and 70℃, 0.5 g of PAN was dissolved in 6 g of DMF solution and stirred to obtain a uniform solution. Then, 1 g of KCuFC powder was added, and the mixture was stirred for another hour to obtain the mixed slurry. The granular adsorbent was prepared in a water-solidified bath. 1 g of the synthesized PAN-KCuFC-PEG was added to 500 mL of simulated rubidium chloride (RbCl) solution, and the mixture was shaken for 4 hours to perform adsorption. After adsorption, the supernatant was collected to analyze the Rb^{+} concentration, and the adsorption efficiency was evaluated as shown in Figure S5.

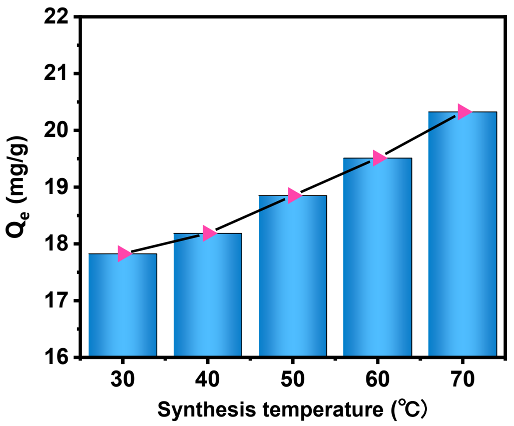

Figure S5. The effect of synthesis temperature on the adsorption performance of granular adsorbent.

4. In a water bath at 70 ℃, 0.5 g of PAN was dissolved in 6 g of DMF, and 0.05, 0.1, 0.15, 0.2, and 0.25 g of PEG were added respectively, mixed and stirred to obtain a uniform solution. Subsequently, 1 g of KCuFC powder was added and the mixture was stirred for an additional hour to obtain a mixed slurry. Then, the granular adsorbent was prepared, ensuring that the mass ratio of PEG to DMF in the composite PAN-KCuFC granular adsorbent was 0.0083, 0.016, 0.025, 0.03, and 0.042. 1 g of the synthesized PAN-KCuFC-PEG was added to 500 mL of simulated rubidium chloride (RbCl) solution, and the mixture was shaken for 4 hours to perform adsorption. After adsorption, the supernatant was collected to analyze the Rb^{+} concentration, and the adsorption efficiency was evaluated as shown in Figure S6.

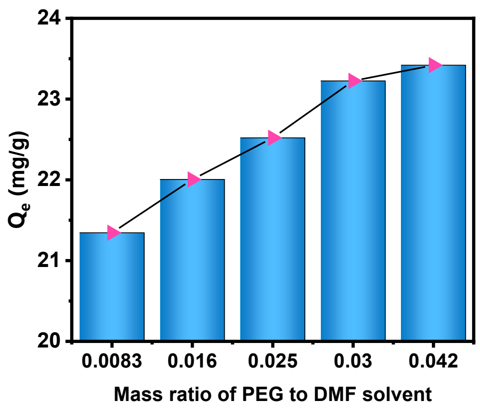

Figure. S6 The effect of the mass ratio of PEG to DMF on the adsorption capacity.

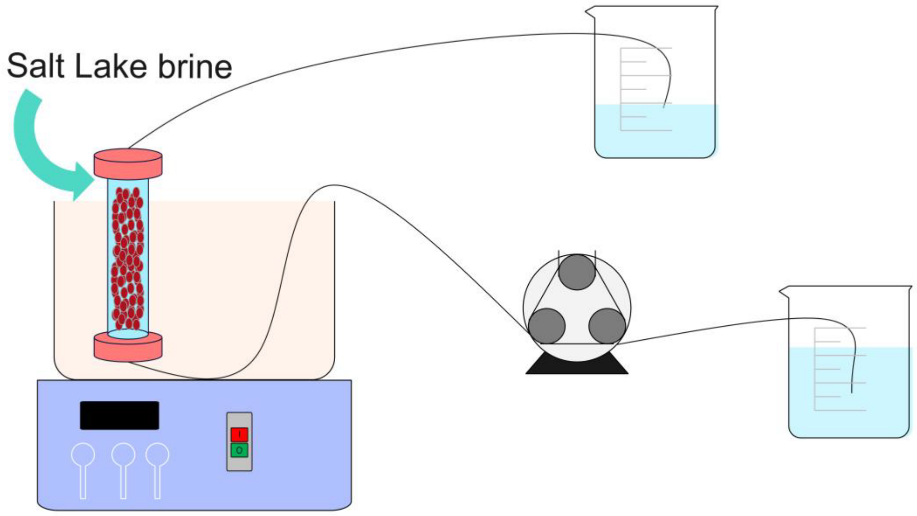

Fig. S7 Schematic diagram of adsorption column adsorption
